# Calcium in the Way: Impella-Supported High-Risk Percutaneous Coronary Intervention in an Elderly Patient with Heart Failure, Progressive Multi-Vessel Coronary Disease, and Prior Iliac Stenting

**DOI:** 10.7759/cureus.104613

**Published:** 2026-03-03

**Authors:** Nina S Appareddy, Bradley Casey, Bhavith Aruni

**Affiliations:** 1 Cardiology, UCHealth Parkview Medical Center, Pueblo, USA; 2 Cardiology, Pueblo Cardiology Associates, Pueblo, USA

**Keywords:** angiographic coronary artery disease, complex percutaneous coronary intervention, elderly patient management, high risk pci, left main coronary artery disease (lmcad), multivessel coronary artery disease (mvcad), primary percutaneous coronary intervention (pci), right coronary artery disease, severe triple vessel cad, short term mechanical circulatory support

## Abstract

We present the case of an 80-year-old male patient with heart failure with reduced ejection fraction and heavily calcified, multi-vessel coronary artery disease involving the distal left main, left anterior descending, and left circumflex arteries, along with a chronic total occlusion of the right coronary artery. He was turned down for surgical revascularization with coronary artery bypass grafting. He initially remained stable on medical therapies and underwent implantable cardioverter defibrillator implantation. He presented back with ventricular fibrillation. Repeat angiography demonstrated progression of his disease. He was planned for high-risk percutaneous coronary intervention supported by a transcatheter micro-axial flow pump. Given his history of peripheral arterial disease (PAD) with prior iliac stenting, vascular surgery was consulted to facilitate large-bore access to initiate support, ultimately deciding on bilateral femoral arterial access.

## Introduction

Coronary artery disease (CAD) remains a major global contributor to morbidity and mortality. Both coronary artery bypass grafting (CABG) and percutaneous coronary intervention (PCI) are established revascularization strategies for multivessel disease. However, determining the optimal approach in patients with severely reduced left ventricular ejection fraction (LVEF) and complex coronary anatomy remains challenging [1,2].

High-risk PCI is commonly characterized by features such as LVEF ≤30-35%, unprotected left main disease, last remaining patent conduit, or anticipated prolonged balloon inflation and calcium modification [3,4]. In such cases, mechanical circulatory support (MCS) may be used to mitigate hemodynamic instability. Transcatheter microaxial flow pumps (Impella; Abiomed, Inc., Danvers, Massachusetts, United States) provide temporary forward flow from the left ventricle to the ascending aorta, unloading the ventricle and maintaining systemic perfusion during complex PCI [5].

Large-bore arterial access (13-14 Fr) required for these devices can present challenges in patients with peripheral arterial disease (PAD), particularly in the presence of calcification, prior stenting, or aneurysmal degeneration [6,7]. Careful pre-procedural vascular imaging and multidisciplinary collaboration are therefore essential.

We present a case illustrating complex decision-making in a patient with severe left ventricular dysfunction, progressive left main disease, arrhythmic instability, and PAD requiring Impella-supported PCI.

## Case presentation

An 80-year-old male patient with hypertension and dyslipidemia presented with several weeks of progressive exertional chest discomfort, dyspnea, and reduced exercise tolerance (New York Heart Association Class II-III). He denied syncope or resting angina.

**Figure 1:**
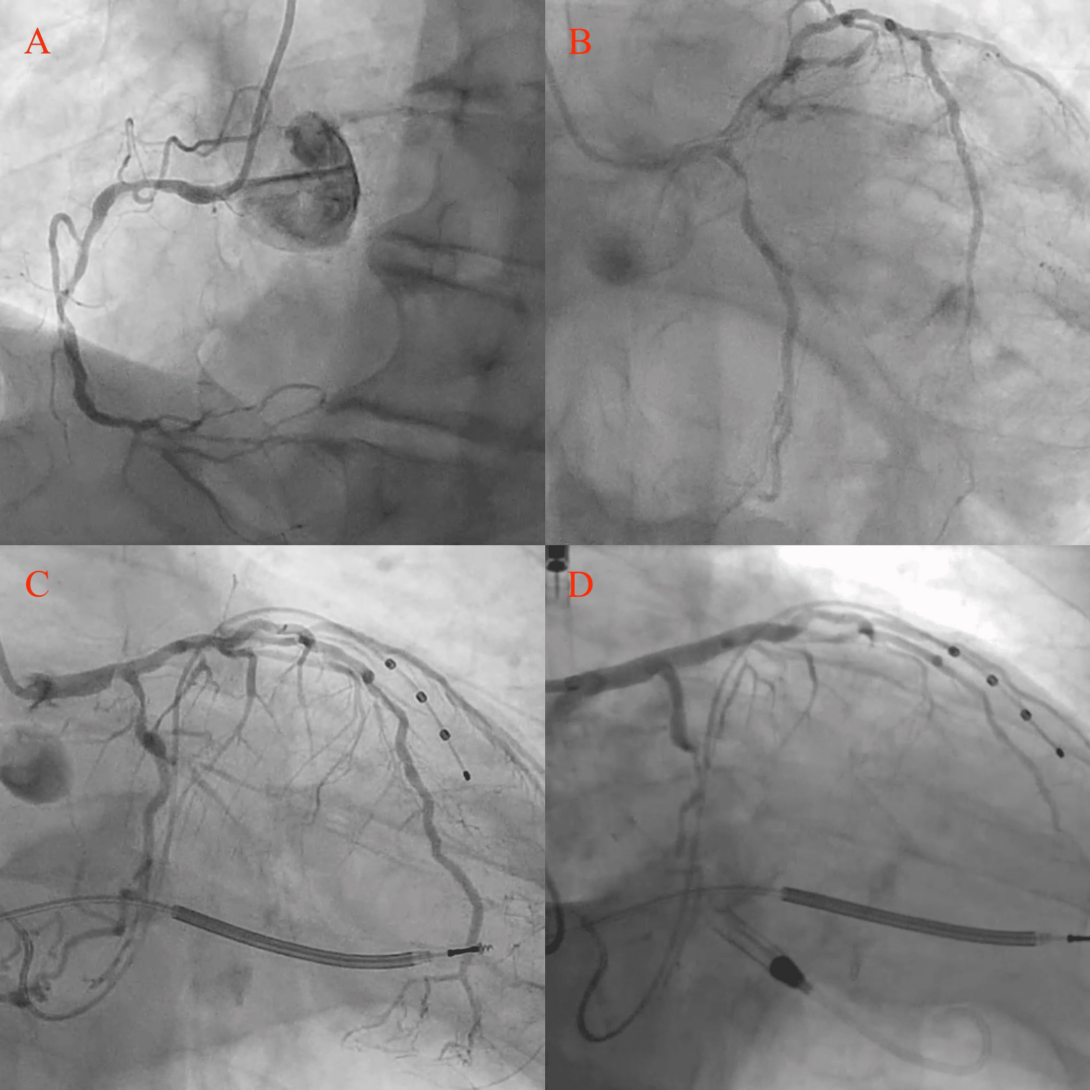
Coronary angiography images (A) chronic total occlusion of the right coronary artery, (B) mild-to moderate disease in the left main coronary artery (LMCA) and obstructive disease to the left anterior descending (LAD) and left circumflex arteries (LCx), (C) repeat angiography following ventricular fibrillation event demonstrating progression of disease, now with obstructive disease in the LMCA along with obstructive disease in the LAD and LCx, and (D) Impella placement for protected percutaneous coronary intervention.

Electrocardiography demonstrated left bundle branch block. Transthoracic echocardiography revealed LVEF 20-25% with global hypokinesis. Pharmacologic stress imaging demonstrated LVEF 18% with fixed inferior and inferolateral defects. Diagnostic coronary angiography via right radial access demonstrated multivessel coronary artery disease involving 60% distal left main (LM) stenosis, 70% proximal left anterior descending artery (LAD) stenosis, 95% proximal left circumflex artery (LCX) stenosis, and a chronic total occlusion (CTO) of the right coronary artery (RCA) (Figure 1A-1B, Videos 1-2).

**Video 1:** Diagnostic coronary angiography showing a chronic total occlusion of the right coronary artery

**Video 2:** Diagnostic coronary angiography of the LMCA system demonstrating multi-vessel coronary artery disease involving 60% distal LM, 70% proximal LAD, proximal 95% LCx, and a chronic total occlusion of the RCA LMCA: left main coronary artery; LM: left main; LAD: left anterior descending artery; LCx: left circumflex artery; RCA: right coronary artery

Cardiothoracic surgery evaluated the patient and declined CABG due to elevated surgical risk. The patient remained clinically stable on guideline-directed medical therapy. Three months later, given persistent LVEF ≤35% and left bundle branch block despite optimal medical therapy, he underwent successful cardiac resynchronization therapy defibrillator (CRT-D) implantation for primary prevention.

**Figure 2:**
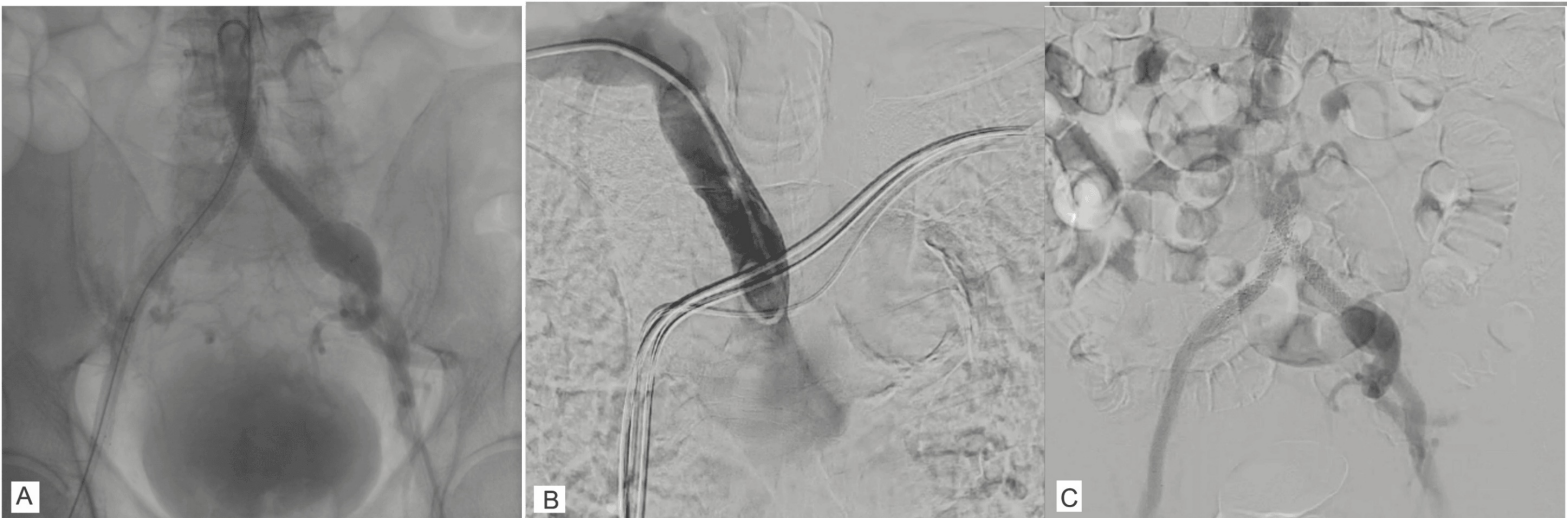
Peripheral angiography images (A) peripheral angiography following bilateral iliac stenting, (B) subclavian angiography, and (C) digital subtraction angiography demonstrating patent bilateral iliac stents.

During follow-up, he reported lifestyle-limiting bilateral lower extremity claudication. Ankle-brachial indices were 0.38 (right) and 0.56 (left). CT angiography demonstrated occlusion of the right common iliac artery extending to the mid external iliac artery with reconstitution via collaterals, and severe stenosis of the left common iliac artery. Given high surgical risk for open bypass, he underwent successful bifurcation stenting of the bilateral common and external iliac arteries (Figure 2A, Video 3).

**Video 3:** Peripheral angiography following successful bifurcation stenting of the bilateral common iliac and external iliac arteries

**Figure 3:**
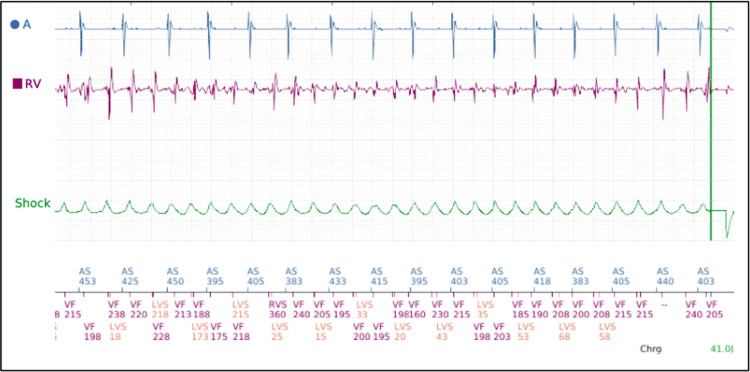
Device interrogation of automated internal cardioverter defibrillator demonstrating ventricular fibrillation event

Approximately six months after initial evaluation, while seated at home, the patient experienced palpitations and presyncope without loss of consciousness. Remote device interrogation demonstrated one unsuccessful anti-tachycardia pacing attempt followed by a 41 J shock that successfully terminated ventricular fibrillation (Figure 3). Repeat coronary angiography demonstrated progression to 90% distal LM stenosis, 90% proximal LAD stenosis, and 95% proximal LCx stenosis, with persistent RCA CTO (Figure 1C, Videos 4-5). Given severely reduced LVEF (20-25%), unprotected distal LM disease, anticipated prolonged calcium modification, and recent VF, high-risk PCI with MCS was planned.

**Video 4:** Repeat coronary angiography demonstrating known chronic total occlusion of the right coronary artery

**Video 5:** Repeat coronary angiography following ventricular fibrillation event demonstrating progression of coronary artery disease, now with 90% stenosis in the distal LMCA, 90% stenosis in the proximal LAD, and 95% stenosis in the proximal LCx. LMCA: left main coronary artery; LAD: left anterior descending artery; LCx: left circumflex artery

Peripheral angiography was performed in anticipation of large-bore access. The right subclavian artery was tortuous and demonstrated aneurysmal degeneration (Figure 2B, Video 6), making axillary access unfavorable. Previously placed iliac stents were patent with adequate luminal diameter to accommodate a 14 Fr sheath, though a left iliac aneurysm was noted (Figure 2C, Video 7).

**Video 6:** Peripheral angiography demonstrating a tortuous right subclavian artery, which is redundant with aneurysmal degeneration

**Video 7:** Peripheral angiography demonstrating previously placed stents in the common and external iliac arteries patent, with a left iliac artery aneurysm noted

After a multidisciplinary discussion with vascular surgery, bilateral ultrasound-guided femoral arterial access was selected with surgical standby in case of vascular complication. Access planning was based on CT and angiographic vessel measurements. No vascular repair was required.

Mechanical circulatory support was initiated via the left femoral artery using an Impella CP device (Figure 1D). The distal LM bifurcation demonstrated severe circumferential calcification (Medina 1,1,1). Rotational atherectomy was performed for superficial calcium modification, followed by intravascular lithotripsy to fracture deep calcium and optimize vessel compliance prior to stent deployment. 

A double-kissing (DK) crush two-stent strategy was selected due to true distal LM bifurcation disease and heavy calcification requiring complete LCx scaffolding. Compared to culotte or T-stenting and small protrusion (TAP), DK crush was favored, given anatomical complexity and operator experience. Drug-eluting stents were deployed as follows: 3.5 × 18 mm to LAD, 3.0 × 20 mm to LCx, and 4.0 × 28 mm to LM. Final angiography demonstrated thrombolysis in myocardial infarction (TIMI) grade 3 flow in both LAD and LCx without residual significant stenosis (Video 8). RCA CTO intervention was deferred due to collateralization and a fixed inferior defect, suggesting non-viable myocardium. No intra-procedural hemodynamic collapse, stroke, major bleeding, or access-site complications occurred.

**Video 8:** Final coronary angiography following successful percutaneous coronary intervention to the left main, left anterior descending, and left circumflex arteries

At the three-month follow-up, the patient remained free of recurrent ventricular arrhythmias or angina. No vascular access complications were observed.

## Discussion

This case illustrates the intersection of severe left ventricular dysfunction (LVEF 20-25%), progressive unprotected left main disease, heavily calcified bifurcation anatomy, and severe PAD with prior iliac stenting. MCS is particularly beneficial in patients with LVEF ≤30-35%, unprotected left main disease, last patent conduit, or anticipated prolonged atherectomy [3,5]. In this case, the recent VF event further supported revascularization under hemodynamic support.

Although axillary access is a viable alternative in PAD [7], subclavian tortuosity and aneurysmal degeneration made it high-risk. Careful CT and angiographic assessment confirmed adequate iliac stent diameter for 14 Fr femoral access, supporting bilateral femoral access with vascular surgery standby.

The combined use of rotational atherectomy and intravascular lithotripsy reflects contemporary calcium modification strategies to optimize stent expansion in severe coronary calcification. Selective peripheral angiography should be considered in patients with known or suspected PAD when large-bore access is anticipated. Early multidisciplinary planning is critical to procedural success.

## Conclusions

Mechanical circulatory support should be strongly considered in patients undergoing PCI with LVEF ≤30-35%, unprotected left main disease, complex bifurcation anatomy, and/or extensive coronary calcification requiring atherectomy. Peripheral vascular imaging should be selectively performed in patients with known or suspected PAD when large-bore access is anticipated. Multidisciplinary planning and careful access assessment can enable safe and successful high-risk PCI even in patients with severe PAD and prior iliac stenting.
